# A COMMUNITY-BASED INTERVENTION TO IMPROVE COGNITIVE FUNCTION IN RURAL APPALACHIAN OLDER ADULTS

**DOI:** 10.1093/geroni/igz038.2039

**Published:** 2019-11-08

**Authors:** Benjamin Katz

**Affiliations:** Virginia Tech, Blacksburg, Virginia, United States

## Abstract

Research suggests that social isolation in Appalachian older adults may be associated with reduced cognitive function. Despite this, few interventions for these individuals incorporate both social and cognitive components in a community-based setting. The “Memory Masterclass” program was developed to address this care challenge, and implemented through an Adult Day Services Center. With 29 community-dwelling older adult participants from western Virginia, the six-week pilot program focused on strategies and practices associated with improving long-term memory or executive function. In addition to the lifestyle-focused curricula, the course included group activities focused on connecting participants to community networks of resources that might enable them to successfully implement lifestyle changes. Findings support the feasibility of implementation with a wider group of rural Appalachian older adults and suggest that individual differences in self-reported memory may be closely tied to improvements following the program.

